# PKCγ promotes axonal remodeling in the cortico-spinal tract via GSK3β/β-catenin signaling after traumatic brain injury

**DOI:** 10.1038/s41598-019-53225-y

**Published:** 2019-11-19

**Authors:** Bo Zhang, Zaiwang Li, Rui Zhang, Yaling Hu, Yingdi Jiang, Tingting Cao, Jingjing Wang, Lingli Gong, Li Ji, Huijun Mu, Xusheng Yang, Youai Dai, Cheng Jiang, Ying Yin, Jian Zou

**Affiliations:** 1 Center of Clinical Research, The Affiliated Wuxi People’s Hospital of Nanjing Medical University, Wuxi, Jiangsu 214023 China; 2 Wuxi Institute of Translational Medicine, Wuxi, Jiangsu 214023 China; 3 Department of Neurology, Shenzhen People’s Hospital, The Second Clinical Medical College of Jinan University, The First Affiliated Hospital of Southern University of Science and Technology, Shenzhen, 518020 China; 4 Department of Neurology, The Affiliated Wuxi People’s Hospital of Nanjing Medical University, Wuxi, Jiangsu 214023 China; 5 Department of Neurosurgery, The Affiliated Wuxi People’s Hospital of Nanjing Medical University, Wuxi, Jiangsu 214023 China

**Keywords:** Molecular neuroscience, Regeneration and repair in the nervous system

## Abstract

Traumatic brain injury (TBI) is a common cause of death and disability. Enhancing the midline-crossing of the contralateral corticospinal tract (CST) to the denervated side of spinal cord facilitates functional recovery after TBI. Activation of the gamma isoform of PKC (PKCγ) in contralateral CST implicates its roles in promoting CST remodeling after TBI. In this study, we deployed loss and gain of function strategies in N2a cells and primary cortical neurons *in vitro*, and demonstrated that PKCγ is not only important but necessary for neuronal differentiation, neurite outgrowth and axonal branching but not for axonal extension. Mechanically, through the phosphorylation of GSK3β, PKCγ stabilizes the expression of cytosolic β-catenin and increase GAP43 expression, thus promoting axonal outgrowth. Further, rAAV2/9-mediated delivery of constitutive PKCγ in the corticospinal tract after unilateral TBI *in vivo* additionally showed that specifically delivery of active PKCγ mutant to cortical neuron promotes midline crossing of corticospinal fibers from the uninjured side to the denervated cervical spinal cord. This PKCγ-mediated injury response promoted sensorimotor functional recovery. In conclusion, PKCγ mediates stability of β-catenin through the phosphorylation of GSK3β to facilitate neuronal differentiation, neurite outgrowth and axonal branching, and PKCγ maybe a novel therapeutic target for physiological and functional recovery after TBI.

## Introduction

Worldwide, traumatic brain injury (TBI) is estimated to impact 50 million people and produce a $400 billion-dollar healthcare burden annually^1^. A leading cause of disability and mortality, TBI is made all the more challenging by its pathophysiological complexity and diversity. Importantly, though functional improvements are well-established during the acute rehabilitation of TBI, up to one third of patients are susceptible to lasting deficits of neuro-motor ability^2^.

The corticospinal tract (CST) plays a critical role in the mammalian motor system, particularly in fine motor functions. Originating predominantly from cortical layer V, various molecular cues are employed in the guidance of CST axons from the cortex through the pyramidal decussation to the contralateral spinal cord^3^. During and after TBI, a variety of injury-responses occur along the CST including microglial activation^4^ and spontaneous axonal regeneration, the latter of which is due to the plasticity of corticospinal projections and is well-associated with functional recovery^5^. Specifically, it has been demonstrated that recovery of motor function is owed (at least in part) to the re-crossing of sprouting CST fibers (from the intact side) through the spinal midline^6,7^, where they are believed to form compensatory motor fiber networks on the denervated side, ultimately contributing to the restoration of motor function after injury. This finding has prompted a wealth of research for growth promoting treatments and strategies that would enhance midline crossing of sprouting CST fibers after injury.

The stimulation of growth promoting signals has been extensively correlated with axonal regeneration after neuronal injury. For instance, the exogenous induction of STAT3 in a rodent model of CST lesion has shown a significant increase in the number of midline crossing fibers that have been specifically shown to target short propriospinal and spinal motor neurons in the intermediate and ventral laminae of the spinal cord^8^. Behavioral strategies such as bilateral movement training have also demonstrated increases in CST midline crossing following TBI in rodents^9^. An alternative approach in promoting midline crossing of sprouting CST fibers has been the target of endogenous mechanisms opposing axonal remodeling. Successful reports have included antagonism of the inhibitory central nervous system (CNS) protein Nogo and its receptor^10,11^ and the inhibitory extracellular chondroitin sulfate proteoglycans^12^, both of which were correlated with functional recovery. Beyond these, many experimental studies have implicated various other therapeutic candidates for CST remodeling including the transmembrane protein MAG^13^ and propyl hydroxylases^14^.

A critical step in the discovery and/or optimization of neural targets for post-injury regeneration and functional recovery is a better understanding the molecular cues which provision functional “detour” networks after injury. Protein kinase C (PKC) isoforms have been implicated across a range of CNS disorders including spinocerebellar ataxia^15^, the hypothyroid brain^16^, and experimental autoimmune encephalomyelitis^17^. The gamma isoform (PKCγ) is found particularly enriched in the nervous system^18^ and has been previously indicted for its role in neuroinflammatory systems and neuropathic pain^19–21^. Its role in the CNS includes developmental pruning of climbing fiber synapses in the cerebellum through the mGluR1-Gq-phospholipase Cβ4-PKCγ signaling pathway^22^. Recently, investigators have shed light on a novel role for PKCγ in the fast and direct interleukin-4 (IL-4) signaling cascade which promotes the phosphorylation of downstream GAP43 and leads to cytoskeletal modifications and axonal growth^23^, indicating its underlying function on cytoskeletal remodeling and axonal repair.

The injury-driven regulation of PKCγ in the CST along with its proposed role in multiple axonal regeneration signaling pathways prompted our investigation of its role in axonal remodeling after TBI. We demonstrated that PKCγ phosphorylation played a crucial role in promoting rehabilitative neuronal differentiation and growth. Moreover, *in vivo* transduction of active PKCγ mutant promotes midline crossing of intact CST fibers and promotes functional recovery after unilateral TBI. Addressing meaningful TBI treatment is of paramount importance due to the evolving and often chronic nature of the disease for many patients. Optimizing molecular strategies for endogenous remodeling after injury could meaningfully alleviate the chronic symptoms of TBI.

## Materials and Methods

### Cell culture and differentiation

The murine neuroblastoma Neuro-2a (N2a) cell line was obtained from the Cell Bank of Type Culture Collection of the Chinese Academy of Sciences (Shanghai, China). Cells were cultured in DMEM (Hyclone, Logan, UT, USA) supplemented with 10% fetal bovine serum (FBS; Biological Industries, Beit-Haemek, Israel). For neuronal differentiation, N2a cells were plated with complete medium (DMEM + 10% FBS) for 12 h to allow adhering, then maintained in differentiation medium (DMEM containing 30% Opti-MEM (Gibco, Grand Island, NY, USA)) for 3–5 days^24^. The differentiation medium was changed every 48 h until harvested. For cells maintained in complete medium, medium was refreshed every 48 h. To evaluate the neuronal differentiation of N2a, cells were immunostained with tubulin, beta 3 class III (Tubulin III, R&D Systems, Minneapolis, MN, USA) and those with neurites extending at least two diameters (of the cell body) were defined as differentiated neuronal cells. Stable N2a cells overexpressing PKCγ-WT, PKCγ-DN, PKCγ-CAT, GSK3β and GSK3β (S9A) were derived from cells infected with the indicated lentiviral constructs and enriched by puromycin selection. Stable N2a cells with depleted PKCγ were derived from Na2 cells infected with lentiviral Cas9-pruo and lentiviral *Prkcg* single guide RNA (sgRNA) using the CRISPR/Cas9 system.

Primary neural stem cells (NSCs) and neurons were cultured as previously described^25–27^. Briefly, the cerebral cortex of E15-E18 BALB/c mouse were isolated, minced and incubated in a solution containing 0.05% trypsin (Gibco) and 0.15% DNase I (Thermo Fisher Scientific, Waltham, MA, USA) at 37 °C for 15 min, followed by triturating and passing through a 70 μm nylon mesh. For cortical NSCs culture, cells were cultured in DMEM/F-12 medium (Gibco) containing 20 ng/ml of basic fibroblast growth factor (bFGF, PeproTech, Rocky Hill, NJ, USA), 20 ng/ml of epidermal growth factor (EGF, PeproTech), N-2 (Gibco) and B-27 (Gibco). For cortical neuron culture, cells were adhered in 37 °C for 15 min to eliminate glial cells and fibroblasts. The supernatant was aspirated and plated on poly-L-lysine (PLL, Sigma-Aldrich, St. Louis, MO) coated dish (Corning, NY, USA) or 14 mm coverslips (Becton Dickinson Labware, Lincoln Park, USA) and maintained in neurobasal media (Gibco) supplemented with B-27 and GlutaMAX (Gibco). For a series of lentivirus infection, acute isolated cells from embryonic cortical tissues were transduced with lentivirus immediately. The natural differentiation of NSCs was according to the previous method^28^. Briefly, NSCs spheres were digested into single-cell suspension using Accutase cell dissociation Reagent (Millipore, Billerica, MA) and subsequently seeded on PLL coated coverslips with NSC medium containing 1% FBS without EGF and bFGF. For Western blot analysis of p-PKCγ in differentiated NSCs and V5–3 treatment of NSCs, a modified neuronal differentiation method was used to improve the differentiation ratio of neurons according to the recommendation of Gibco website. Briefly, the digested NSCs were seeded on PLL coated coverslips or dishes with NSC culture medium for 2 days, and changed the medium to neuronal culture medium (Neurobasal medium with B27 and GlutaMAX) for another 5 days. The neuronal culture medium was changed every 2 days.

### Vector construction

The CRISPR/Cas9 system was applied to deplete PKCγ accordingly. Briefly, lentiviral Cas9-pruo and lentiviral *Prkcg* single guide RNA (sgRNA) were derived from Genechem (Shanghai, China). The sgRNA sequence targeting mouse *Prkcg* was 5′-ATATGGATCTCATCCGACGT-3′; 5′-CTGTGTGGTCCACACCGCAA-3′. A general sgRNA was used as a negative control (NC):5′-CGCTTCCGCGGCCCGTTCAA-3′. The target sequence was inserted into GV371 lentiviral vector (Genechem).

For PKCγ expressing constructs (PKCγ-WT, PKCγ-CAT and PKCγ-DN), cDNA was amplified by PCR from the plasmids obtained from the Addgene plasmid depository (Addgene plasmids 21236, 21238 and 21239) and verified by DNA sequencing. The sequences were cloned into the lentiviral vector GV230 (Genechem) fused with green fluorescent protein (GFP). The human GSK-3β wild-type and GSK-3β constitutively active mutant (GSK3β S9A) cDNA was obtained from the Addgene plasmid depository (Addgene plasmids 14753 and 14754) and inserted into the lentiviral vector GV348 (Genechem) fused with a HA-tag, for the infection of primary cortical neurons, cDNA was inserted into the lentiviral vector GV230 (Genechem) fused with GFP-tag. The adenoviral rAAV2/9-PKCγ-CAT-GFP cDNA was amplified from the PKCγ-CAT plasmid and its control rAAV2/9-GFP was established by Obio Technology (Shanghai, China).

### Peptide synthesis

The PKCγ-specific inhibitory peptide, V5-3 (targeting PKCγ amino acids 659–664: CRLVLAS) was designed in a previously published study^29^. For cell and blood brain barrier permeation, the 11 amino acid residues of TAT-PTD (YGRKKRRQRRR) were derived from the HIV TAT protein and were conjugated at the N-terminus via a cysteine–cysteine bond^30^. A peptide V5-3s with scrambled sequence (CRVLALS) was deployed as a negative control. Peptides were synthesized by GenScript (Nanjing, China). In N2a differentiation, V5-3 (20 nM) was added simultaneously with 30% OM; in neurite outgrowth and branching assay, N2a cells were incubated in 30% OM for 2 days to allow differentiation and neurite formation, then V5-3 (20 nM) was added into the 30% OM for another 3 days.

### Immunofluorescence analysis

Immunofluorescence procedures were performed as previously reported^31^. Coverslips with cells or tissue sections were labeled with primary antibodies overnight at 4 °C. Antibodies used included rabbit anti-MAG (Thermo Fisher Scientific), mouse anti-Tubulin III (R&D Systems), rabbit anti-Tubulin III (Abcam, Cambridge, MA, USA), mouse anti-GFAP (Millipore, Billerica, MA, USA), rabbit anti-p-PKCγ (Abcam), mouse anti-MAP2 (Thermo Fisher Scientific), mouse anti-β-catenin (Abmart, Shanghai, China), mouse anti-Nestin (Abcam), rabbit anti-Rip (a gift from Dr. Scott R. Whittemore, University of Louisville) and mouse anti-hemagglutinin (HA)-tag (Abmart). Cell nuclei were counterstained with Hoechst 33342 (Invitrogen). The staining was visualized using an EVOS FL microscope (Life technology, Gaithersburg, MD, USA) or a laser scanning confocal microscope (Leica Microsystems GmbH, Mannheim, Germany).

### Immunohistochemistry (IHC)

The procedures of immunohistochemical (IHC) staining were performed according to a previously reported protocol^32^. Briefly, sections were rinsed with PBS (0.1 M, pH = 7.4) and incubated with an avidin-biotinylated peroxidase complex (ABC) followed by the immunoperoxidase diaminobenzidine tetrahydrochloride (DAB) method according to the manufacturer’s instructions (Invitrogen). The primary antibody, mouse anti-SMI-31 antibody (Millipore) was applied. Sections were mounted on glass coverslips and observed using an EVOS FL microscope. The primary mouse IgG (CoWin Bio, China) was used to confirm the specificity of the IHC labeling.

### Western blot analysis

Western blot assays were followed according to an established protocol^32^. Briefly, dissected cerebral cortex tissues, cervical spinal cord tissues or cells were lysed in RIPA lysis buffer (Cell Signal Technology, Beverly, MA). Protein concentrations were determined using a BCA assay (Pierce, Roclford, IL, USA) and equal amounts were loaded onto 10% polyacrylamide gel (Bio-Rad, Hercules, CA, USA) and separated by SDS-PAGE. Samples were then transferred to PVDF membranes, blocked in 5% nonfat dried milk (Becton, Dickinson and Company, Franklin Lakes, NJ, USA), and incubated overnight at 4 °C with primary antibodies. Antibody labelling was detected by incubation with horseradish peroxidase-labelled anti-rabbit/mouse secondary antibody (Proteintech, Wuhan, China). Protein was visualized using chemiluminescence (Millipore). Antibodies evaluated by Western blot included rabbit anti-p-PKCγ (Abcam), rabbit anti-GAP43 (Abcam), mouse anti-β-Catenin (Abmart), rabbit anti-phospho-GSK-3β (Ser9) (Cell Signal Technology), mouse anti-GAPDH (Thermo Fisher Scientific), mouse anti-β-actin (Thermo Fisher Scientific), mouse anti-HA-tag (Abmart), and mouse anti-GFP (Abcam).

### Immunoprecipitation and ubiquitination assays

Immunoprecipitation and ubiquitination evaluation were performed as previously described^32^. In brief, cells were treated with MG132 (Abcam) at a final concentration of 20 μm for 4 h before collection. Whole-cell lysates were prepared in RIPA buffer and precipitated using protein A/G beads (Abmart) with β-Catenin antibody (Abmart). Precipitated products were assayed by immunoblot (IB) analysis with anti-Ub antibody (Abcam) or β-Catenin antibody.

### Neurite outgrowth assay

To measure the neurite outgrowth of N2a cells, cells were immunostained on glass coverslips with different fluorescence markers and visualized using an EVOS FL microscope. The percentage of cells with neurites extending at least two times the diameter of the cell body was calculated using Image-Pro Plus software (Media Cybernetics, Silver Springs, MD, USA).

### Neurite length assay

Neurons cultured for 5 days were double immunofluorescence stained with anti-Tubulin III and anti-MAP2 antibodies. The fiber indicated Tubulin III positive and MAP2 negative was marked as axon. For lentivirus transduced neurons, only GFP^+^ cells were measured and statistical analyzed. For neurite length assessment of N2a cells, the length of longest neurite and number of neurites extending at least two diameters of the cell body were examined. The length of axon (or longest neurite) of N2a was measured using Image-Pro Plus software from at least 30 cells per condition. The average neurite length was used for statistical analysis.

### Cell proliferation and apoptosis assays

Proliferation of N2a cells was assayed by cytometry analysis of kFlour647 Click-iT EdU kit (KeyGen Biotech, Nanjing, China) according to the accompanying protocol. Briefly, N2a cells were cultured in 10% FBS for 12 h, and changed to 30% OM or 10% FBS for another 4 days. EdU (10 μΜ) was added to culture medium 2 hours before collection. Cells were washed and stained according to the protocol and detected immediately by flow cytometry (BD FACS Canto II). Cell growth was monitored by Cell Counting Kit-8 (Bimake, Houston, TX, USA) according to the instruction. Cell apoptosis was assayed using a commercial Annexin V-Alexa Fluor 647/PI apoptosis detection Kit (Fcmacs Biotech, Nanjing, China). N2a cells were cultured for 4 days in 30% OM or 10% FBS. Then, cells were harvested and stained according to the instruction. The percentages of Annexin V and/or PI-positive cells were determined by flow cytometry. Annexin V single positive and Annexin V/PI double positive cells were counted as apoptotic cells. Each experiment was repeated at least 3 times.

### Traumatic brain injury and tissue preparation

Four-week-old female BALB/c mice were purchased from the Changzhou Cavens Laboratory Animal Co. Ltd. (Changzhou, China). The mice were anesthetized with Avertin (2.5%, 0.2 ml/20 g; Sigma-Aldrich) and were placed in a stereotactic frame adapted for mouse. The TBI procedure was performed as described previously^33^, briefly the skull was exposed and a circular craniotomy (4.5 mm radius) was made midway between the bregma and lambda, and 2.5 mm lateral to midline over the left hemisphere. Next, mice were subjected to injury via a 1.0 mm impact depth using an electromagnetic impactor (RWD, China). For detecting the overall p-PKCγ expression in spinal cord after TBI, cervical spinal cord segments (C2-C7) were derived from one case at one time point. For detecting CST loss after TBI, spinal cord sections derived from 14 days post-injured cervical spinal cords from 3 individual animals per group were immunostained with SMI-31, MAG and p-PKCγ. For AAV injection, a total of 18 mice were assigned randomly to 3 groups: sham (n = 6), rAAV2/9-GFP (n = 6) and rAAV2/9-PKCγ-CAT-GFP (n = 6). Immediately after TBI operation, the skull in the right hemisphere was carefully uncovered, and the AAV was injected into a total of 4 sites (0.5 ul per site over a 3–5 minute time period) using a 10 μl NanoFil microsyringe tipped with a 36 G micropipette. Coordinates were 1.0 mm lateral, 0.5 mm deep to the cortical surface, and +1.0, +0.5, −0.2, and −0.7 mm with respect to bregma. In the sham-operated control group, the animals received the same surgical procedure without the impact portion. Five weeks post-injury, the mice were given an overdose of Avertin and perfused with 0.9% saline followed by 100 ml 4% paraformaldehyde (PFA, Sigma-Aldrich). Following perfusion, the brain and entire spinal cord were carefully removed, post-fixed, and equilibrated in 30% sucrose (Sigma-Aldrich) in PBS. Frozen coronal sections of brain and cervical spinal cord (C2-C7) or horizontal sections of the cervical spinal cord (C2-C7) were processed serially (20 μm thick) on a cryostat (CM950, Leica Microsystems, Buffalo Grove, IL). The sections were stored at -20 °C. For immunostaining, 150 μm apart and spanning the entire sectioned spinal cords were selected randomly (10 sections per individual mice). Additionally, for the analysis of the right cortex of targeted area after AAV injection, another five mice per group were used after three weeks post-injury. All animal care and handling were performed in accordance with the National Institutes of Health’s Guide for the Care and Use of Laboratory Animals. All study procedures were approved by the Institutional Review Board of Nanjing Medical University (Permit number: KYLLH2018006). No mice were excluded from scoring. All animal experiments were conducted in a double-blinded manner.

### CST fibers’ quantification

The quantification of the crossing axons was followed to the established method^9,34^. The IOD of GFP-positive fibers crossing from intact side to the denervated side were calculated. The data were derived from 10 sections, which were selected randomly from 150 μm apart and spanning the entire sectioned C2-C7 spinal cords. To normalize of differences in the tracing efficiency of the individual animals, the number of crossing fibers was divided by the total IOD of main CST fibers in the intact gray matter.

### Foot-fault test

To test sensorimotor function after TBI, the foot fault test was carried out at 7, 14, 21, 28 and 35 days after TBI. The mice were allowed to walk on a grid^35^. A foot fault was defined as a paw fall or slip between the wires with each weight-bearing step^36^. A total of 50 steps and faults of the right forelimb and hindlimb were recorded for each run of the test. Scoring was performed by two independent research team members who were blinded to the animal’s treatments. The mice were trained at day 7, 6, 5 and 1 before the surgery. A total of 3 runs are recorded for each animal.

### Adhesive removal test

The adhesive removal test for sensorimotor function was also performed 35 days after TBI according to a previously described protocol^33^. Briefly, a 3 mm × 4 mm adhesive strip was pasted onto right forepaw. Animals were observed in a transparent box. Mouse behavior was monitored by a camera. The time-to-contact and the time-to-remove the tape were noted within a maximum 3 min session for each assessment. The time-to-contact was defined as the time at which the mouse reacted to the tape by shaking the right paw or brining the paw to its mouth. The time-to-remove was defined as the point when the tape was removed by mouth. If the animal failed to remove the tape within a 3 min session, the time-to-remove was noted as 3 min.

### Statistical analysis

Data are expressed as mean ± SD. The difference between two independent samples or multiple groups were determined by Student’s *t-*test or one-way analysis of variance (ANOVA) followed by a Newman Keuls’ multiple comparison test respectively, with statistical significance at *p* < 0.05. SPSS 16.0 package (IBM) and GraphPad Prism 6.0 software (GraphPad Software) were used to carry out all statistical analyses and data graphing, respectively.

## Results

### PKCγ is activated in CST projections from the uninjured cortex after unilateral TBI

**Figure 1:**
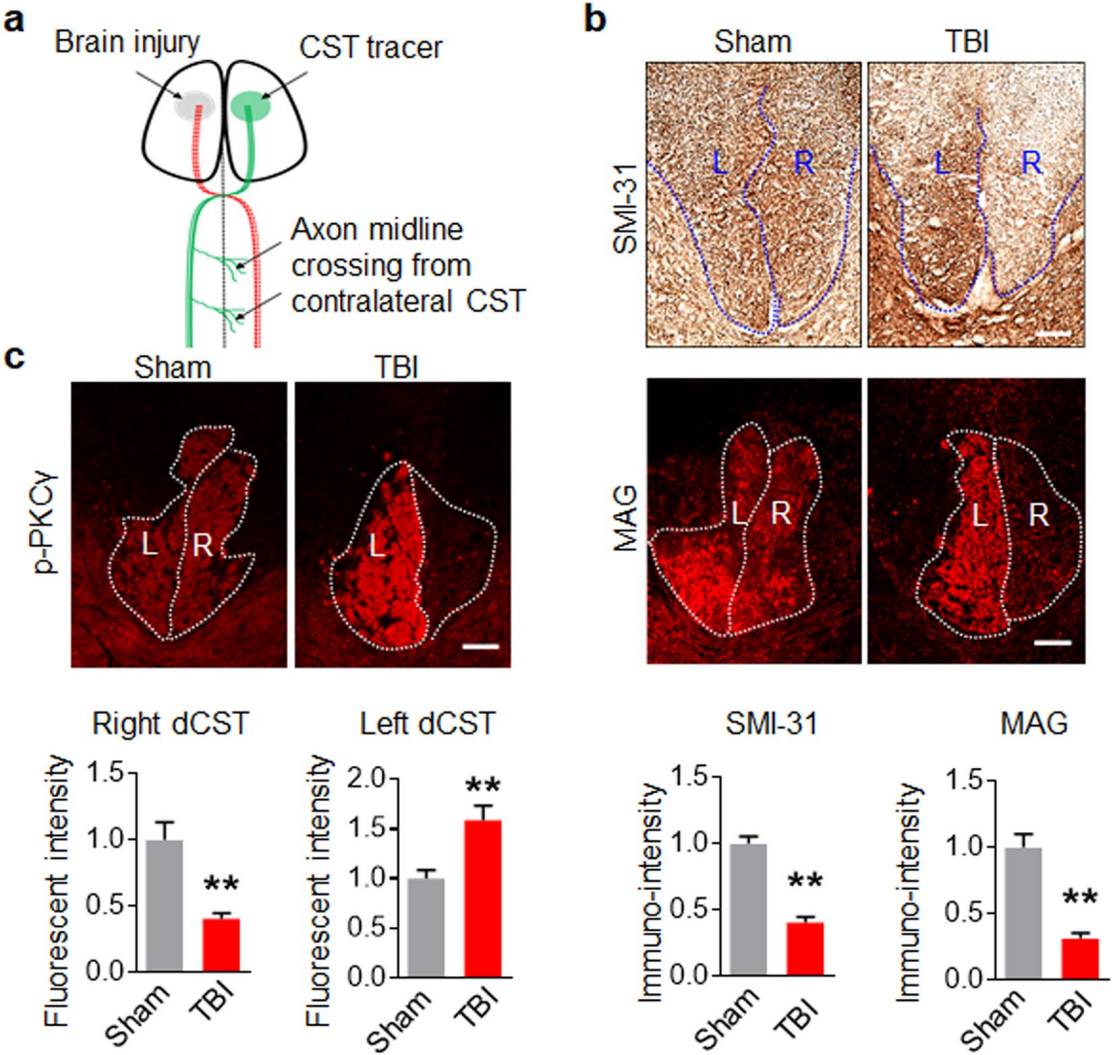
PKCγ is phosphorylated in intact CST after unilateral TBI. (**a**) Simplified schematic illustration of unilateral TBI, CST degeneration and plasticity. Unilateral TBI causes cortical injury which affects CST degeneration (red dotted lines). CST from the contralesional, intact cortex sprouts axons that cross the midline into the denervated spinal cord after TBI (green lines). This sprouting can be anterogradely labelled by CST tracer. (**b**) Unilateral TBI of left cortex destroyed the right dorsal CST (dCST) in the cervical spinal cord. Cervical spinal cord sections derived from 2 weeks post-injured mice were immunostained with SMI-31 or MAG antibody. The lower panel showed the relative immune-intensity of right dCST to the contralateral dCST. (**c**) Immunofluorescence (IF) staining showed an increase of p-PKCγ expression in left dCST and a decrease in the right dCST in the cervical spinal cord after TBI. The immune-intensity of p-PKCγ in the left or right dCST was compared to homolateral dCST in Sham spinal cords. Data are expressed as mean ± SD (n = 3 animals in each group) and compared by Student’s *t*-test (**p < 0.01 vs Sham). Scale bars, 50 μm.

We first established a unilateral TBI model (Fig. 1a) and determined whether unilateral TBI could destroy its descending CST completely. The cervical spinal cords were stained with SMI-31 and MAG which label axon and myelin respectively, 2 weeks post-injury. SMI-31 and MAG immunoreactivity were present bilaterally in the dorsal CST (dCST) of the cervical spinal cord in sham-operated mice. On the other hand, SMI-31 and MAG immunoreactivity were mostly disappeared in the right dCST originating from the injured left cortex (Fig. 1b), indicating that unilateral TBI resulted in a destruction of the CST descending from the injured cortex. Based on this result, the overall phosphorylated PKCγ (p-PKCγ) was measured in the cervical spinal cords from one case at indicated times after TBI. As shown in Supplemental Fig. S1, p-PKCγ was increased 3 days post-injury and remained highly expressed up to 21 days post-injury. Further, immunofluorescence staining showed that p-PKCγ was bilaterally expressed in the dCST of the cervical spinal cord in sham-operated mice, while a loss of p-PKCγ was observed in the right dCST after unilateral TBI. Moreover, an increase in p-PKCγ was found in the left dCST, indicating that unilateral TBI induced an activation of PKCγ in CST descending from the intact cortex as well (Fig. 1c).

### Activity of PKCγ is associated with neuronal differentiation and growth

**Figure 2:**
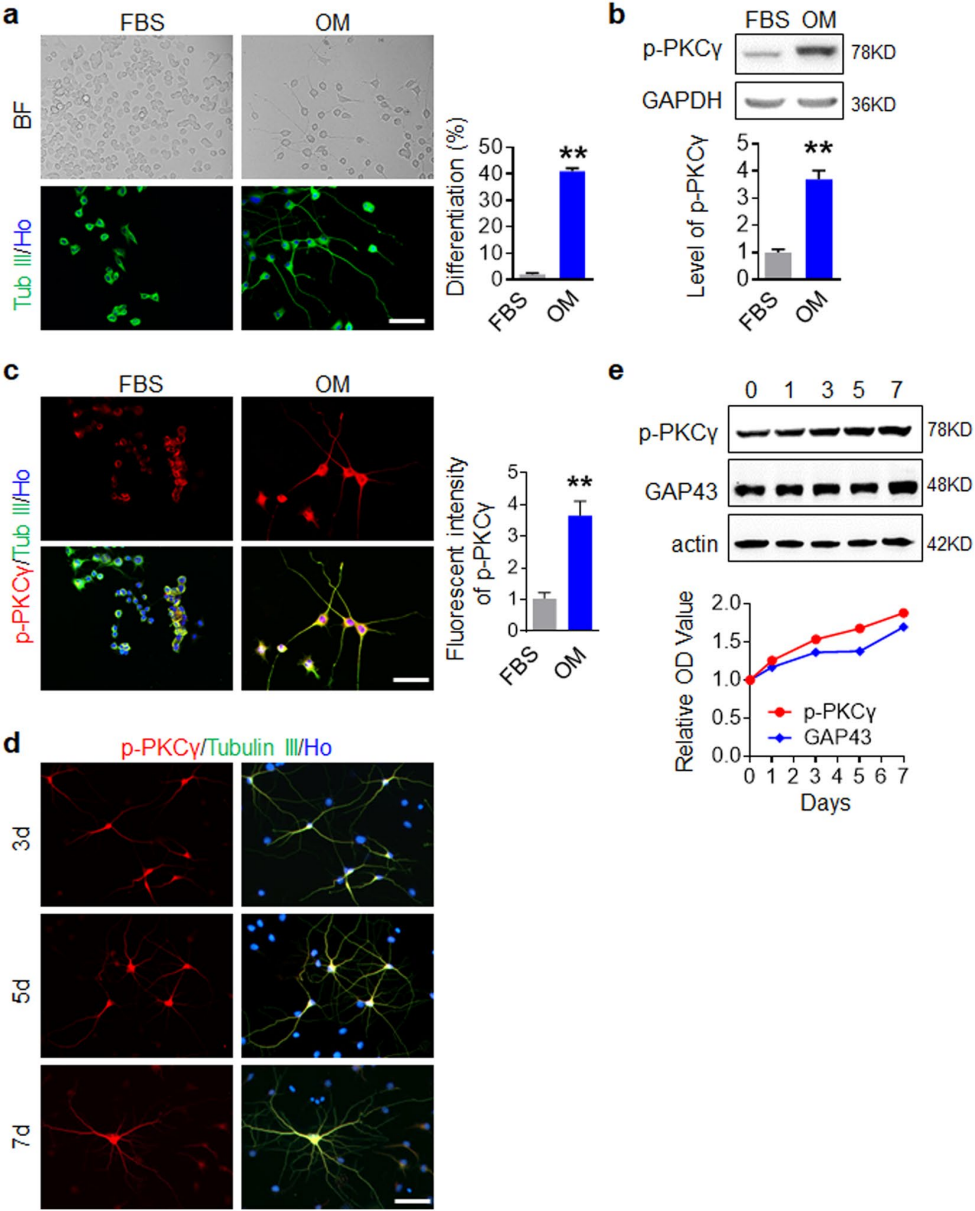
Activity of PKCγ is associated with neuronal differentiation and growth. (**a**) 30% OM induced neuronal differentiation of N2a. Neuronal differentiation of N2a was shown by bright field (BF) and Tubulin III (Tub III) immunostaining. (**b**,**c**) PKCγ was activated in differentiated N2a induced by 30% OM. N2a cultured with 30% OM for 4 days were collected and p-PKCγ was analyzed by Western blot (**b**) or double IF staining (**c**). Hoechst (Ho) labeled the nuclei. Data are expressed as mean ± SD (n = 3 individual experiments in each group) and compared by Student’s *t*-test (**p < 0.01 vs FBS). (**d**,**e**) PKCγ was activated in growing neurons. Neurons cultured at the indicated times were analyzed by double IF staining (**d**) or Western blot (**e**). GAP43 was used to monitor the neuronal growth. Scale bars, 50 μm.

To explore the potential function of PKCγ in the spontaneous plasticity of CST axons after TBI, we first determined whether PKCγ is involved in neuronal differentiation and growth in our model. According to the previous report, the mouse neural crest-derived N2a cell line has the ability to differentiate into neurons by serum deprivation and/or treatment with other stimuli and is widely used to study neuronal differentiation, neurite growth, synaptogenesis and signaling pathways^37^. We established a neuronal differentiation model of N2a using DMEM + 30% Opti-MEM (30% OM) as a differentiation inducing-supplement according to a reported method^24^. As shown in Fig. 2a, cells treated with 10% FBS displayed a round shape without neurite extension, whereas neurite extensions were observed in 30% OM treated cells under phase contrast microscopy and according to Tubulin III immunostaining. The spontaneously differentiation ratio of N2a cells was lower than 2% when maintained in 10% FBS while up to 50% when treated with 30% OM. 30% OM treatment resulted in a lower proliferation (Supplemental Fig. S2a,b), but without significant apoptosis (Supplemental Fig. S2c). The time lapse of same field images further confirmed that 30% OM induced neuronal cells derived from N2a can proliferate (Supplemental Fig. S2d). Western blot assay, as well as double immunostaining of p-PKCγ and Tubulin III further showed that p-PKCγ was significantly increased in 30% OM treated cells, suggesting that p-PKCγ is activated in differentiated N2a cells (Fig. 2b,c). For neural stem cells (NSCs) differentiation, we used an established method to induce spontaneous differentiation^27^. As shown in Supplemental Fig. S3a, NSCs (Nestin positive) differentiated into either of three types of neural cells under NSC medium containing 1% FBS without EGF and bFGF: neurons (Tubulin III positive), astrocytes (GFAP positive) and oligodendrocytes (Rip positive). p-PKCγ was predominantly expressed in neurons (Supplemental Fig. S3b). To verify whether PKCγ was activated in differentiated neurons, neuronal culture medium was used to improve the neuronal differentiation ratio of NSCs. It showed that p-PKCγ was increased in neuronal differentiated NSCs (Supplemental Fig. S3c), indicating that p-PKCγ is associated with neuronal differentiation. We next examined the expression of p-PKCγ in maturing neurons. Primary embryonic mouse cortical neurons cultured for the indicated length of time demonstrated that p-PKCγ was continuously expressed in mature neurons (Fig. 2d). Further, Western blot assay substantiated that p-PKCγ was constitutively activated in growing neurons (Fig. 2e). These results indicated that PKCγ is activated during neuronal differentiation and is likely associated with neuron growth.

### Activation of PKCγ is essential for neuronal differentiation

**Figure 3:**
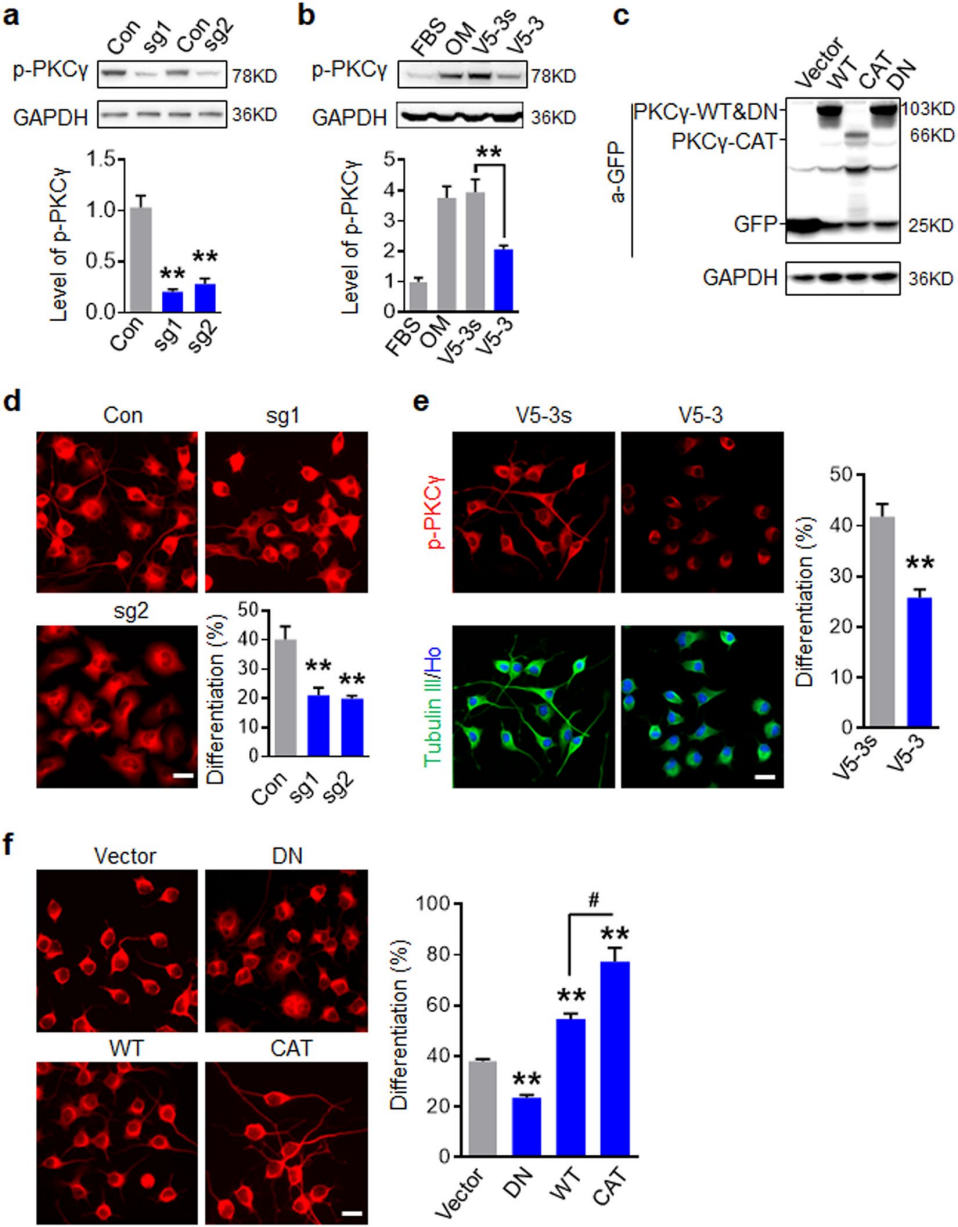
Activation of PKCγ is essential for neuronal differentiation of N2a. (**a**) Western blot analysis of PKCγ depletion in N2a cells mediated by CRISPR/Cas9. (**b**) Western blot analysis showed that V5-3 inhibited PKCγ activation induced by 30% OM in N2a. (**c**) Western blot analysis indicated various PKCγ lentiviral constructions were expressed in N2a. (**d**) PKCγ depletion inhibited neuronal differentiation induced by 30% OM. N2a were immunostained with Tubulin III antibody and the percent of differentiated N2a cells with GFP^+^ was quantified. (**e**) V5-3 (20 nM) attenuated neuronal differentiation of N2a. (**f**) Activation of PKCγ facilitated neuronal differentiation of N2a. N2a cells were infected with indicated PKCγ constructs and induced differentiation. For d and f, the percent of differentiated cells with GFP^+^ was quantified in right panel. All data are expressed as mean ± SD (n = 3 individual experiments in each group) and compared by one-way ANOVA (**a**,**b**,**d**,**f**) or Student’s *t*-test (**e**) (**p < 0.01, ^#^p < 0.05 vs indicated group). Scale bars, 20 μm.

To investigate whether PKCγ activation is a key contributor of neuronal differentiation, we established series constructs that targeted PKCγ expression or activity (Fig. 3a–c). First, we used CRSIPR/Cas9 mediated knock out to examine whether PKCγ deletion affected neuronal differentiation of N2a cells (Fig. 3a). To evaluate the neuronal differentiation of N2a, cells were immunostained with Tubulin III and those with at least one neurite extending over 2 diameters of the cell body were classified as neuronal cells. As shown in Fig. 3d, neuronal differentiation was significantly inhibited in cells treated with sgRNAs targeting PKCγ. Further, N2a cells treated with 30% OM and V5-3 peptide (20 nM), a PKCγ antagonist^29^, for four days indicated that the increase of phosphorylated PKCγ induced by 30% OM was inhibited by V5-3 treatment (Fig. 3b). As expected, cells treated with V5-3 consequently showed a lower ratio of neuronal differentiation than control peptide-treated cells (Fig. 3e). Meanwhile, no significant cell growth inhibition was found in 30% OM culture condition with V5-3 and V5-3s incubation (Supplemental Fig. S4). Importantly, PKCγ inhibition by V5-3 impaired the neuronal differentiation of NSCs, but did not affect astrocyte and oligodendrocyte differentiation (Supplemental Fig. S3d). Next, we established a series of PKCγ lentivirus constructions (Fig. 3c) including wide-type (PKCγ-WT), dominant negative type (PKCγ-DN) and constitutively activated type (PKCγ-CAT). In N2a differentiation assay with 30% OM, PKCγ-DN expressing cells showed a decreased differentiation ratio, while PKCγ-WT expressing cells showed an increased differentiation ratio and the expression of PKCγ-CAT got the highest differentiation potential (Fig. 3f). These results revealed that activation of PKCγ is not only a correlate but an essential factor for neuronal differentiation.

### Activation of PKCγ facilitates growth of axonal branches

**Figure 4:**
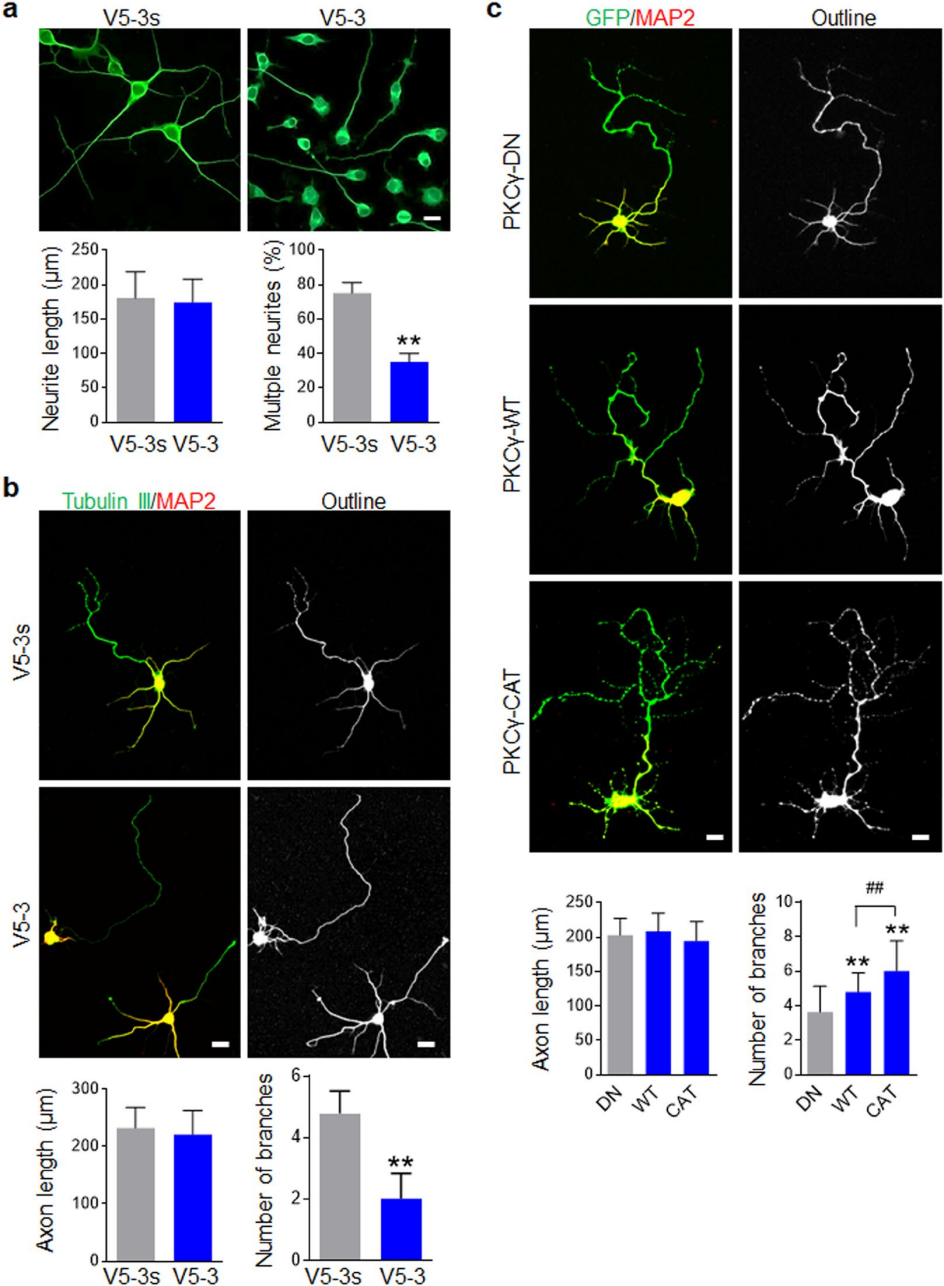
Activation of PKCγ facilitates branch growth of axon. (**a**) V5-3 suppressed neurite outgrowth of N2a, but did not inhibit neurite length. Neuronal differentiation of N2a was induced by 30% OM for 2 days and cells were incubated with or without V5-3 (20 nM) for an additional 3 days. The cells were immunostained with Tubulin III antibody. The length of longest neurite (defined as >2 × diameter of the cell body) was measured and percent of cells with multiple neurites was quantified. Data are expressed as mean ± SD (n = 3 individual experiments in each group) and compared by Student’s *t*-test (**p < 0.01). (**b**) V5-3 inhibited axonal outgrowth. Neurons cultured for 5 days were double stained with Tubulin III (Green) and MAP2 (Red). Neurons were outlined, the axon length and number of axonal branches were quantified (lower panel). Data are expressed as mean ± SD (n = 50 neurons of three individuals in each group) and compared by Student’s *t*-test (**p < 0.01, ^##^p < 0.01 vs indicated group). (**c**) Constitutive activation of PKCγ facilitated axonal branch outgrowth. Neurons infected with the indicated construct were cultured for 5 days and immunostained with MAP2 antibody (Red). Axons were determined by GFP^+^/MAP2^−^ and its branch number and length were quantified. Data are expressed as mean ± SD (n = 30 GFP^+^ neurons of three individuals in each group) and compared by one-way ANOVA (**p < 0.01, ^##^p < 0.01 vs indicated group). Scale bars, 20 μm.

To investigate whether PKCγ specifically regulates neurite outgrowth, we determined whether PKCγ inhibition impacts neurite outgrowth. N2a cells were treated with 30% OM for 2 days to allow differentiation and neurite formation. Cells were further co-incubated with V5-3 in 30% OM for another 3 days and subjected to Tubulin III immunostaining. As shown in Fig. 4a, cells treated with the control peptide (V5-3s) formed an average of two or more neurites per cell, while those treated with V5-3 mostly formed a single neurite with fewer branches. Interestingly, V5-3 had no effect on the length of neurite. Then, we examined whether V5-3 executed inhibitory action on axon outgrowth. With the aid of MAP2/Tubulin III double IF labeling, axons (MAP2^−^/Tubulin III^+^) were identified in 5 day cultured primary neurons. Similar to N2a cells, V5-3 treatment inhibited axonal branch outgrowth, but had no effect on axon length (Fig. 4b). To further confirm the role of PKCγ in regulating neurite and axonal outgrowth, neurons were transduced with the indicated PKCγ lentiviral construct followed by 5 days culture. Cells were immunostained with MAP2 to identify axon. As shown in Fig. 4c, PKCγ-WT overexpression resulted in increased axonal branch outgrowth, while PKCγ-CAT overexpression further enhanced this effect. Neither PKCγ-WT nor PKCγ-CAT demonstrated an effect on axon length, suggesting that PKCγ is a regulator for axonal branch outgrowth, rather than a factor for axon extension.

### PKCγ induces axonal outgrowth by increasing the stability of β-catenin

**Figure 5:**
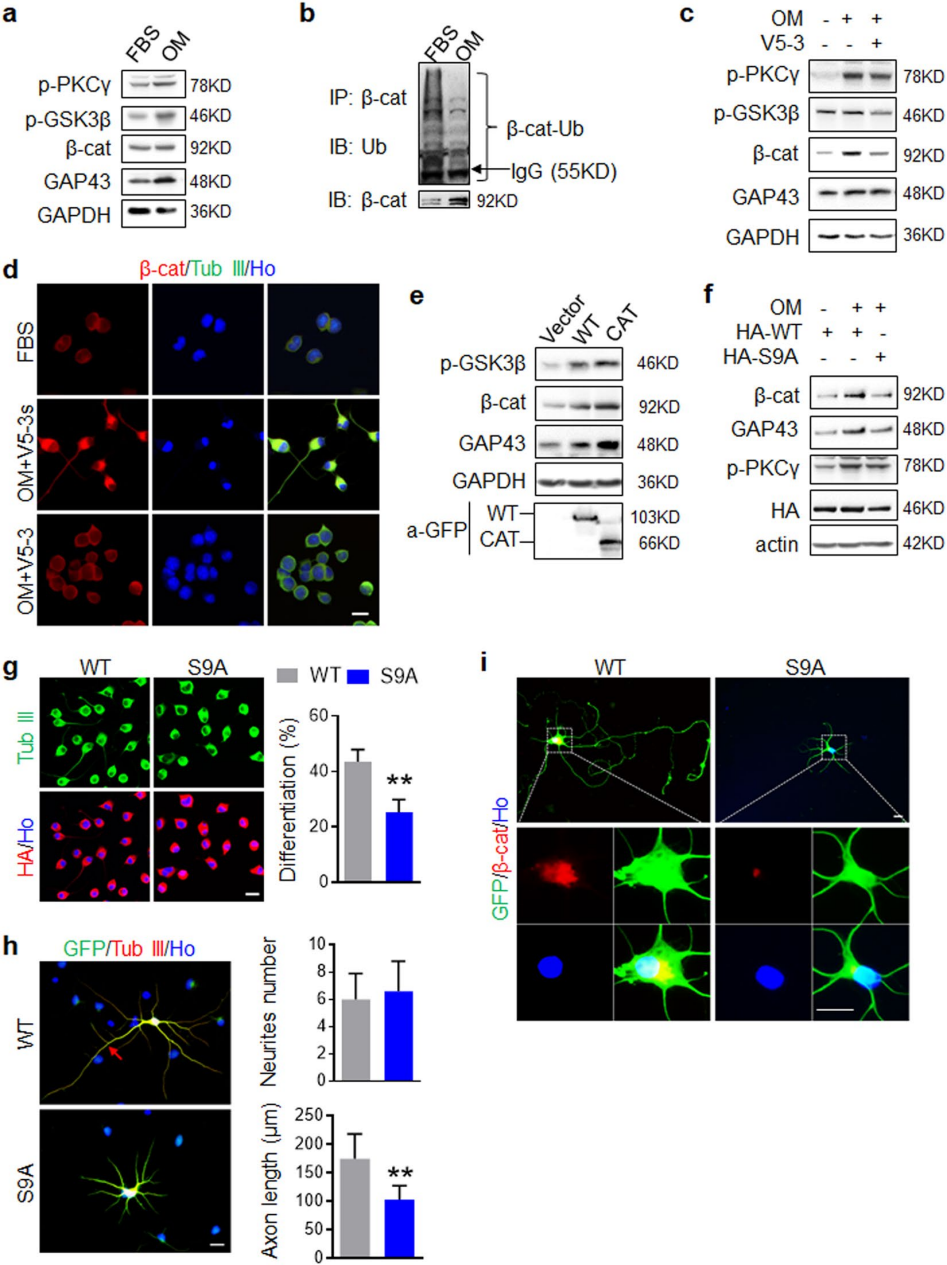
PKCγ induces axonal outgrowth via stabilization of β-catenin. (**a**) 30% OM decreased GSK3β activity and increased β-catenin expression in N2a cells. N2a cells were cultured with 30% OM for 3 days to initiate neuronal differentiation and the indicated proteins were examined. (**b**) OM increased β-catenin stability by inhibiting ubiquitin degradation of β-catenin. N2a were cultured with 30% OM for 3 days, followed by IP with anti-β-catenin and IB with the indicated antibodies. (**c**) PKCγ inhibition increased GSK3β activity and decreased β-catenin expression in differentiated N2a. N2a were cultured in 30% OM with or without V5-3 and the indicated proteins were analyzed by Western blot. (**d**) V5-3 inhibited β-catenin upregulation induced by 30% OM. N2a were cultured with 30% OM for 4 days and followed by double IF staining. (**e**) Constitutive activation of PKCγ increased β-catenin expression and inhibited GSK3β activity in N2a. N2a overexpressing PKCγ-WT or PKCγ-CAT cells were cultured in 30% OM and the indicated proteins were analyzed by Western blot. (**f**) Constitutive activation of GSK3β attenuated β-catenin upregulation in N2a induced by 30% OM. N2a overexpressing GSK3β-WT or GSK3β-S9A were cultured in 30% OM for 4 days and the indicated proteins were analyzed by Western blot. (**g**) Constitutive activation of GSK3β attenuated neuronal differentiation of N2a. Data are expressed as mean ± SD (n = 3 individual experiments in each group) and compared by Student’s *t*-test (**p < 0.01 vs WT). (**h**) Constitutive activation of GSK3β inhibited axonal elongation. Neurons transfected with GSK3β-WT or GSK3β-S9A were cultured for 5 days. The number of neurites and the length of the longest neurite were quantified. Data are expressed as mean ± SD (n = 30 GFP^+^ neurons of three individuals in each group) and compared by Student’s *t*-test (**p < 0.01 vs WT). (**i**) Constitutive activation of GSK3β attenuated β-catenin accumulation in the cell body toward to the axon. Neurons transfected with GSK3β-WT or GSK3β-S9A were cultured for 5 days and IF staining with β-catenin antibody was performed. Scale bars, 20 μm.

To assess the signaling involved in PKCγ- induced neuronal differentiation and neurite outgrowth, the expression level of phosphorylated GSK3β (p-GSK3β) and β-catenin were examined by Western blot. As shown in Fig. 5a as well as in Supplemental Fig. S5a, 30% OM treatment resulted in a higher level of GSK3β phosphorylation at Ser9, β-catenin, GAP43 and p-PKCγ in N2a cells. As GSK3β forms a complex with APC/Axin leading to ubiquitination and proteasomal degradation of β-catenin^38^, we performed a β-catenin ubiquitin co-immunoprecipitation assay to confirm that the upregulation of β-catenin was derived from GSK3β inactivation-induced protein stability in N2a cells (Fig. 5b, Supplemental Fig. S5b). Application of V5-3 revealed that 30% OM induced GSK3β/β-catenin alterations are indeed regulated by PKCγ activity (Fig. 5c, Supplemental Fig. S5c). Once GSK3β is phosphorylated, stabilized β-catenin is translocated to the nucleus where it binds TCF4, releasing co-repressors and recruiting additional co-activators to target gene. Considering the important roles of β-catenin/TCF4 in regulating NSCs self-renewal and differentiation, we further investigated the cellular localization of β-catenin after experimental treatment. Immunostaining indicated that β-catenin was mainly expressed in non-nuclear components in normal N2a cells and the cellular distribution of β-catenin, including nuclear translocation, was not altered in differentiated cells (Fig. 5d). In PKCγ-WT and PKCγ-CAT- expressing N2a cells, we further confirmed that PKCγ is a regulator of GSK3β/β-catenin (Fig. 5e, Supplemental Fig. S5d). Based on these findings, the functions of GSK3β in neuronal differentiation and neurite outgrowth were examined. We established a constitutively active form of GSK3β (GSK3β-S9A) and a GSK3β-WT for stable expression in N2a cells via lentivirus transduction. Western blot analysis showed that GSK3β-S9A inhibited 30% OM-induced β-catenin upregulation without affecting p-PKCγ (Fig. 5f, Supplemental Fig. S5e), indicating that β-catenin upregulation in differentiated N2a is directly attributable to GSK3β inhibition. As expected, GSK3β-S9A overexpression significantly inhibited neuronal differentiation of N2a (Fig. 5g). Meanwhile, GSK3β-WT infected primary neurons exhibited normal morphology with one long neurite (identified as axon, arrow marked) and several smaller neurites while GSK3β-S9A infected neurons showed irregular morphology lacking even one prominent neurite. Neurites quantification further indicated that GSK3β constitutive activation inhibited axonal growth or determination, without affecting neurites number (Fig. 5h). Similar to the observations in N2a, the expression of β-catenin was declined in neurons bearing GSK3β-S9A (Fig. 5i), though the cytoplasmic (toward the axon) expression of β-catenin was not altered by the treatment. Collectively, these data revealed that PKCγ-induced neuronal differentiation and axonal outgrowth operate through the inhibition of GSK3β activity and the consequent stabilization of β-catenin in the cytoplasm.

### rAAV2/9 mediated PKCγ-CAT transduction promotes midline-crossing of intact CST and functional recovery after unilateral TBI

**Figure 6:**
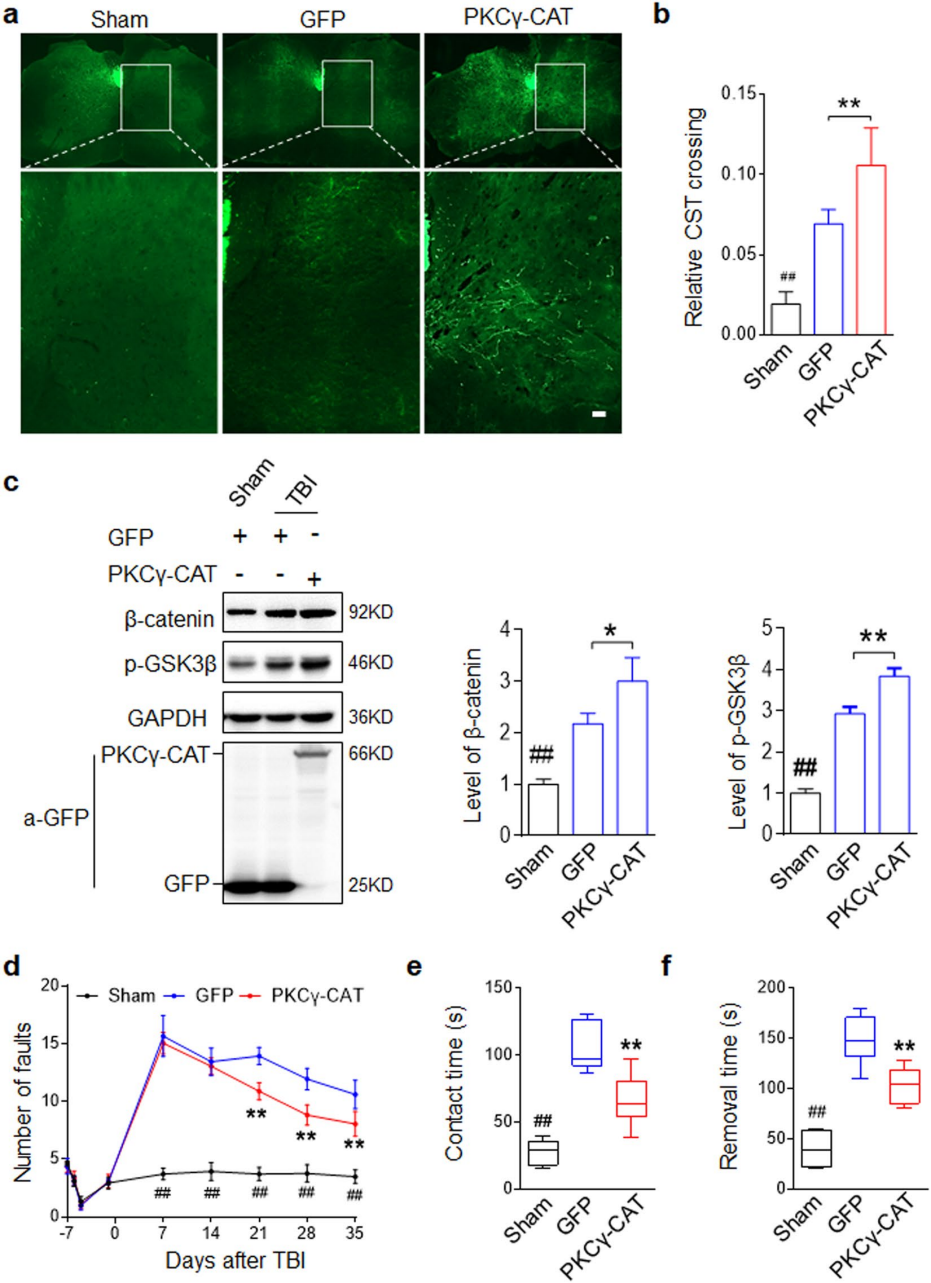
rAAV2/9 mediated PKCγ-CAT delivery promotes midline-crossing of intact CST and functional recovery after unilateral TBI. (**a**) rAAV2/9 mediated PKCγ-CAT delivery promoted midline-crossing of intact CST after unilateral TBI. rAAV2/9 virus was injected into the contralateral cortical motor sensory region immediately after unilateral TBI. Cervical spinal cord sections showed the rAAV2/9-GFP marked dorsal CST and midline-crossing CST fibers. (**b**) Quantification of midline-crossing CST fibers. Data are expressed as mean ± SD (n = 6 animals in each group) and compared by one-way ANOVA (^##^p < 0.01 vs Sham, **p < 0.01 vs GFP). Scale bars, 40 μm. (**c**) PKCγ-CAT delivery promoted β-catenin and inhibited GSK3β activity in right cortex. Mouse right cortical tissue were collected three weeks after TBI and indicated proteins were evaluated by Western blot. GAPDH served as a loading control. Data are expressed as mean ± SD (n = 3 animals in each group) and compared by one-way ANOVA (*p < 0.05, **p < 0.01, ^##^*p* < 0.01 vs indicated group). (**d**) Foot-fault training and test at indicated days after TBI. Number of fall steps from the right limbs were counted and compared. (**e,f**) Adhesion removal test at 35 days after TBI. Time-to-contact (**e**) and time-to-remove the adhesion tape from the right paw (**f**) were compared. For d-f data are expressed as mean ± SD (n = 6 animals in each group) and compared by one-way ANOVA (^##^*p* < 0.01 vs Sham, **p < 0.01 vs GFP).

Based on our *in vitro* characterization of PKCγ during neuronal differentiation and axonal outgrowth, we next investigated whether PKCγ could serve as a potential target to promote CST plasticity after TBI. For this purpose, we deployed a neuronal expression virus, the adeno-associated virus (rAAV2/9-hSyn-GFP) expressing green fluorescent protein (GFP) under a Synapsin I^39^ promoter. Based on this virus, we established a PKCγ-CAT-GFP fusion expressing construct. The AAV virus was injected into right sensorimotor cortex opposing the left impacted cortex (Supplemental Fig. S6a). With the aid of a GFP tag, we observed that the rAAV2/9 virus successfully infected the sensorimotor cortex after 3 weeks post-injury (Supplemental Fig. S6b). Western blot analysis of the cortex tissue additionally indicated that rAAV2/9 successfully delivered the gene into the targeted area (Fig. 6c). Immunofluorescence staining indicated that the rAAV2/9 construct was specifically expressed in cortical neurons, excluded in astrocytes (Supplemental Fig. S6c). In the cervical spinal cord, we detected the GFP signal in the left dorsal CST descending from right cortex (Supplemental Fig. S6d). The horizontal sections revealed that rAAV2/9 infected cortical neurons can transmit the ectopic protein along the CST fibers (Supplemental Fig. S6e). These data indicated that the rAAV2/9 virus can be used not only as a CST tracer, but as a CST-specific gene therapy carrier. Fibers sprouting from the intact CST across the midline to the denervated side of the spinal cord are necessary for functional recovery after unilateral TBI^7,9^. To determine whether PKCγ-CAT delivery enhanced CST plasticity, we examined the ratio of midline-crossing fibers into the denervated side in the cervical spinal cord (C2–C7) 35 days after injury (Fig. 6a,b). We observed that the number of GFP-labeled midline crossing fibers increased spontaneously following injury (Fig. 6a,b; Sham versus GFP, ^##^*p* < 0.01). Meanwhile, the PKCγ-CAT group showed a significantly higher number of midline-crossing CST fibers compared with Sham controls (^##^*p* < 0.01) or the GFP group (^**^p < 0.01). Further, we observed that TBI induced a decrease in GSK3β activity and concurrent increase in β-catenin expression in the mouse cortex, an effect that was further enhanced by PKCγ-CAT delivery after 3 weeks post-injury (Fig. 6c). To evaluate the functional consequences of PKCγ-CAT delivery on motor and sensorimotor function after TBI, we utilized two behavioral tests, the foot-fault test and adhesion removal test. These evaluations have been reported as sensitive indicators for sensorimotor impairment in rodents^40,41^ previously. The foot fault test was administered to Sham, GFP, and PKCγ-CAT-expressing animals 7, 14, 21, 28, 35 days after treatment. Sham (uninjured) animals demonstrated a consistent, low number of foot faults throughout the entire study period while GFP and PKCγ-CAT groups demonstrated a significantly higher number of foot faults (Fig. 6d). While no difference was observed between the GFP and PKCγ-CAT group at the 7- and 14-day mark, PKCγ-CAT-treated animals made significantly fewer foot faults 21-35 days after experimental treatment indicating recovery of limb function (^##^*p* < 0.01 Sham versus GFP or PKCγ-CAT group, **p < 0.01 PKCγ-CAT versus GFP group). Similarly the adhesion removal test demonstrated a consistent contact (sensory) and removal (motor) response time at 35 days while both GFP and PKCγ-CAT groups demonstrated delayed contact and removal times (Fig. 6e,f; Sham versus GFP or PKCγ-CAT group, ^##^*p* < 0.01). PKCγ-CAT treated groups were able to detect and remove adhesive strip faster than their GFP counterparts (^**^p < 0.01), indicating an improved stimulus-directed movement.

## Discussion

TBI induces an inflammatory and regenerative post-injury CST response spontaneously. Sprouting of collaterals from the intact side can cross the anatomical midline and compensate denervated CSTs on the injury side, often restoring motor function. PKC isoforms are dynamically altered in experimental models of brain injury^42^. Although PKCγ is a CST associated molecule, its response and role in CST after TBI is largely unknown.

In the present study, we found that phosphorylated PKCγ is upregulated during TBI in spinal cords on the uninjured side. Previous characterizations of PKCγ in the spinal cord are typically noted in CST axons and, though limited, have reported upregulation following induction of visceral pain^43^, and spinal contusion^44^. In contrast, it is reportedly decreased in the CST in EAE^17^ and in the lumbar SC in a model of SCI^45^. Here we present a novel characterization for the expression of PKCγ in the spinal cord in a mouse model of TBI.

PKCγ has displayed diverse biological roles during development and disease, from pruning synapses in the postnatal cerebellum^46^ to regulating nociception during peripheral nerve injury^20^. Its neuroprotective role in various brain injury models^47,48^ and neuron-specific expression^18^ has hinted at a biological role in neural rehabilitation. Here we demonstrated in an N2a cell line of neuronal differentiation that phosphorylated PKCγ is increased with GAP43 and continuously and specifically expressed in differentiating neurons. Moreover, using a CRISPR/Cas9 mediated KO and peptide antagonist of PKCγ we confirmed that not only is the isoform involved in neuronal differentiation but it is necessary for the critical function *in vitro*. While classic PKC isoforms have been correlated with neuronal differentiation^49,50^ and indicted in essential phosphorylation of ERK/MAPK for neurite outgrowth^51,52^ and photoreceptor differentiation^53^, we present the first evidence of the essential nature of the gamma isoform for neuron differentiation.

Because axonal growth is a critical event in neuronal regeneration and sprouting post injury, we explored whether PKCγ may specifically contribute to axon outgrowth and extension. Using the N2a cell line and primary neurons, we confirmed that PKCγ activation is essential for neurite outgrowth and axonal branching, but not axonal extension. Various PKC isoforms have been tied to neurite outgrowth post injury, including in the sciatic nerve^54^ and retinal neurites^55^. More substantive evidence that PKCγ plays a role in neurite outgrowth comes from previous reports of PKC inhibition, which may diminish growth cone formation, differentiation, and neurite outgrowth^56,57^. In our findings PKCγ did not alter axon length in any experimental modality despite previous implications of PKC signaling in conferring developmental neurite elongation in motor neurons and organotypic culture^58^. Interestingly, a directly targeted molecule of PKCγ, glycogen synthase kinase 3β (GSK3β), has been shown to regulate various microtubule growth and transport molecules^59^ and is important for maintaining neuronal polarity-a critical factor of axon elongation^60^. It stands to reason that though preservation and upregulation of the GSK3β-targeting PKCγ may promote neurite outgrowth overall, it may play a more complex role in the mechanisms conferring downstream neuronal polarity and axonal elongation.

To investigate the mechanisms of PKCγ in axonal outgrowth and branching further, we assayed the direct PKCγ target GSK3β, a serine/threonine kinase that is phosphorylated and specifically inactivated by PKCγ and other PKC isotypes^61^. Under normative conditions, GSK3β plays a role in the rapid turnover of free β-catenin through complex formation with AXIN1/2, APC, and CKI α. Specifically, GSK3β is responsible for sequential phosphorylation of β-catenin which marks the target for subsequent ubiquitination and proteasomal degradation^62^. We found that phosphorylated GSK3β at the serine 9 residue is increased in differentiated neurons concomitant with increases in β-catenin. The application of a PKCγ peptide inhibitor as well as the constitutive active PKCγ mutant, further confirmed that the GSK3β/β-catenin signal was mediated by PKCγ *in vitro* and *in vivo*. PKCγ was also found to mediate downstream GAP43, a prominent phosphoprotein in axon/synapse growth^63^.

To confirm that β -catenin was under direct regulation of GSK3β in this model, we used a construct of constitutive GSK3β expression which downregulated β-catenin without disturbing PKCγ. Consequently, the construct inhibited axonal growth without affecting neurite number. Collectively, our results paint a specific and sequential signaling cascade for PKCγ-mediated axonal growth and re-iterate that although PKCγ plays a robust role in neurite outgrowth, the GSK3β/β-catenin pathway specifically mediates axonal outgrowth. Whether inactivation of GSK3β inhibits or promotes axonal regeneration is highly contended. Our results here resonate with previous descriptions of enhanced axonal regeneration^64,65^ though, (among others) a genetic manipulation strategy to achieve constitutively active GSK3 have reported the opposite effect^66^. This indicates that the role of GSK3 and its downstream kinase activity may be multi-dimensional and sensitive to methodological strategy. Ultimately, upregulation of GAP43 and GSK3β-induced upregulation of β-catenin provide sufficient evidence that PKCγ-induced regulation can lead to the activation of the dynamic and well-established Wnt/β-catenin and GAP43 axonal growth elements^67,68^. In the future, however, it would be essential to confirm the activation of subsequent gene targets and their unique contributions to neurite and axonal growth effect in our system.

To explore whether the PKCγ-mediated molecular signals guiding neurite and axonal growth could translate into therapeutic action *in vivo*, we deployed an rAAV2/9 mediated GFP-PKCγ construct under the Synapsin I promoter into the contralateral sensorimotor cortex, which successfully and specifically delivered the product into cortical neurons. Moreover, the construct was demonstrably expressed along the CST as observed across horizontal cervical sections. As predicted, CST fibers descending from transfected cortical neurons displayed sprouting to the denervated side, indicating successful midline crossing up to 35 days after injury. In the same model, cortical reductions in GSK3β and upregulation of β-catenin were observed. As previously mentioned, midline crossing of intact CST collaterals to the denervated side are a spontaneous and effective re-organizing strategy of cortico-spinal motor neurons after injury. Across multiple injury types, midline crossing is correlated with functional motor recovery^8,9,14^ and has in one study been demonstrated as a requirement for motor recovery^7^. Here, the sustained enhanced midline crossing CST collaterals were additionally correlated with improvements in functional recovery after TBI. Specifically, improvements were detected across limb function and sensorimotor response 21–35 days after injury and treatment. However, total rehabilitation was not demonstrated in either test as function remained significantly impaired compared to uninjured controls. Our rAAV2/9 delivery mechanism not only shows a successful mean to track PKCγ or other gene products in the CST, but offers a novel, therapeutic target for enhanced midline crossing and functional recovery in TBI.

Some limitations of this study include the lack of characterization of PKCγ-signal mediated gene target alterations which underlie neurite and axonal growth, particularly Wnt/β-catenin and GAP43 targets, many of which have previously outlined for neuronal differentiation and axonal growth^69,70^. Although this study uncovered that active PKCγ facilitates axonal branching sprouting, the underlying mechanism of PKCγ activation and the polarity of midline crossing characterization after TBI has not been fully understood. The possibility that the compensation derived from TBI induced intrinsic signaling cannot be excluded. However, the stimulation from the end of axons is more likely cause of PKCγ activation. Our unpublished data uncovered that SDF1 was elevated in denervated side of spinal cord and its receptor, CXCR4 was increased in the crossing CST fibers after TBI, suggested that chemokine and its receptor maybe a key factor. Clarifying the cause of p-PKCγ elevation after injury is also expected to conducing spontaneous remolding and regeneration after TBI.

In summary, we presented PKCγ as a regulator of neuronal differentiation and neurite outgrowth *in vitro* and begin to resolve the signaling cascades that confer its action in neurite and axonal growth. Moreover, we demonstrate that virus-mediated constitutive expression of the protein kinase results in enhanced CST midline crossing and partial functional recovery in a mouse model of TBI. These findings poise PKCγ as a novel therapeutic target for physiological and functional repair after TBI as well as shed light on the molecular mechanisms of PKCγ during the TBI injury response.

## Supplementary information

Supplementary Information
