# Supplementary material for: PKCγ promotes axonal remodeling in the cortico-spinal tract via GSK3β/β-catenin signaling after traumatic brain injury
Source: Sci Rep. 2019 Nov 19;9:17078. doi: 10.1038/s41598-019-53225-y (PMC6863826; doi:10.1038/s41598-019-53225-y)
Supplement: Supplementary file 1 — Supplementary Information [file 41598_2019_53225_MOESM1_ESM.docx]

**PKCγ promotes axonal remodeling in the cortico-spinal tract via GSK3β/β-catenin signaling after traumatic brain injury**

**Running Title:** PKCγ promotes CST remodeling after mouse brain injury

Bo Zhang ^1,2^, Zaiwang Li ^3,4^, Rui Zhang ^1,5^, Yaling Hu ^1,2^, Yingdi Jiang ^1,2^, Tingting Cao ^4^, Jingjing Wang ^1,2^, Lingli Gong ^1,2^, Li Ji ^1,2^, Huijun Mu ^1,2^, Xusheng Yang ^1,2^, Youai Dai ^1,2^, Cheng Jiang ^1,2^, Ying Yin ^1,2,*^, Jian Zou ^1,2,*^

^1^ Center of Clinical Research, The Affiliated Wuxi People's Hospital of Nanjing Medical University, Wuxi, Jiangsu, 214023, China.

^2^ Wuxi Institute of Translational Medicine, Wuxi, Jiangsu, 214023, China.

^3^ Department of Neurology, Shenzhen People's Hospital, The Second Clinical Medical College of Jinan University, The First Affiliated Hospital of Southern University of Science and Technology, Shenzhen 518020, China.

^4^ Department of Neurology, The Affiliated Wuxi People's Hospital of Nanjing Medical University, Wuxi, Jiangsu, 214023, China.

^5^ Department of Neurosurgery, The Affiliated Wuxi People's Hospital of Nanjing Medical University, Wuxi, Jiangsu, 214023, China.

These authors contributed equally to this work: Bo Zhang, Zaiwang Li, Rui Zhang

* **Correspondence to:** Jian Zou, Center of Clinical Research, The Affiliated Wuxi People's Hospital of Nanjing Medical University, 299 Qingyang Road, Wuxi, 214023, China. Tel: +86-510-85350368; E-mail: [zoujan@njmu.edu.cn](mailto:zoujan@njmu.edu.cn); Ying Yin, Center of Clinical Research, The Affiliated Wuxi People's Hospital of Nanjing Medical University, 299 Qingyang Road, Wuxi, 214023, China. Tel: +86-510-85350363; E-mail: [yinying83@njmu.edu.cn](mailto:yinying83@njmu.edu.cn).

**
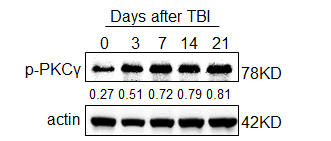
**

**Supplemental Figure S1. PKCγ was activated in cervical spinal cord after TBI.**

The phosphorylation of PKCγ (p-PKCγ) at indicated times post-TBI was analyzed by Western blot. Actin served as a loading control. The relative expression of p-PKCγ to actin was listed.


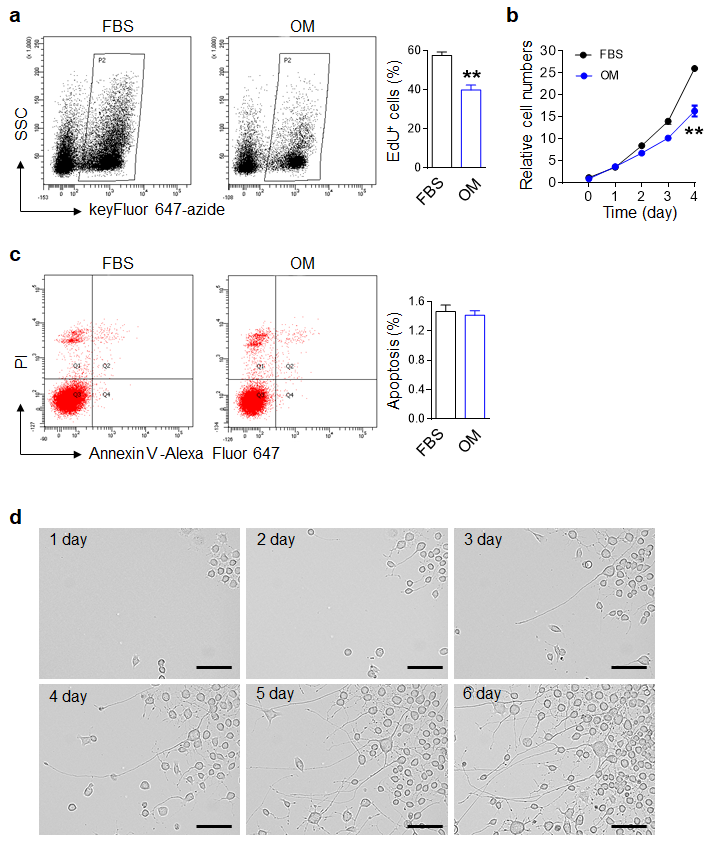


**Supplemental Figure S2. 30% OM treatment impaired N2a cell proliferation, but not induce apoptosis.**

(a,b) Cell proliferation and growth of N2a were detected by flow cytometry analysis of EdU labeling (a) and CCK-8 (b) in FBS or 30% OM at 4 days. (c) Apoptosis of N2a assayed by flow cytometry analysis of Annexin V/PI. Data are expressed as mean ± SD (n = 3 individual experiments in each group) and compared by Student’s *t-*test (**p < 0.01 vs FBS). (d) 30% OM induced neuronal differentiation of N2a in the same field vison of bright field for 6 days, Scale bars, 100 μm.


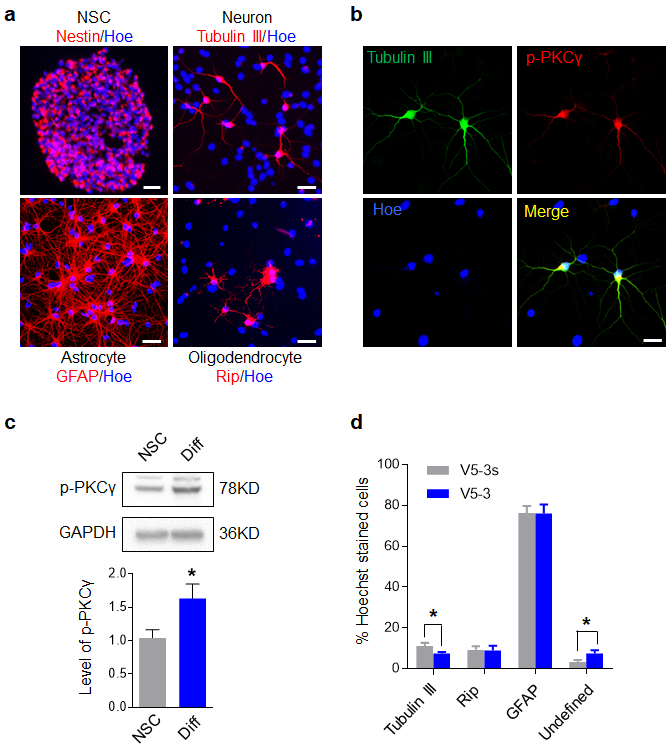


**Supplemental Figure S3. Activation of PKCγ is essential for neuronal differentiation of NSCs.**

(a) Natural differentiation of mouse neural stem cells (NSCs). The NSCs sphere and its differentiated cells were stained with the indicated antibodies. (b) p-PKCγ was mainly expressed in neurons differentiated from NSCs. Differentiated cells from NSCs were double-stained with Tubulin III and p-PKCγ. (c) p-PKCγ was increased in differentiated cells from NSCs. Data are expressed as mean ± SD (n = 3 individual experiments in each group) and compared by Student’s *t-*test (*p < 0.05 vs NSC). (d) p-PKCγ inhibition impaired neuronal differentiation of NSCs but not affect astrocyte and oligodendrocyte differentiation. Data are expressed as mean ± SD (n = 3 individual experiments in each group) and compared by Student’s *t-*test (*p < 0.05 vs V5-3s). Scale bars, 50 μm.


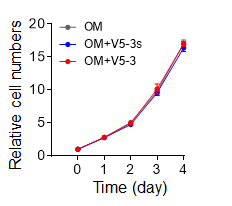


**Supplemental Figure S4. V5-3 did not inhibit N2a cell growth in differentiation medium with 30% OM.**

Cell growth assayed by CCK-8.

**
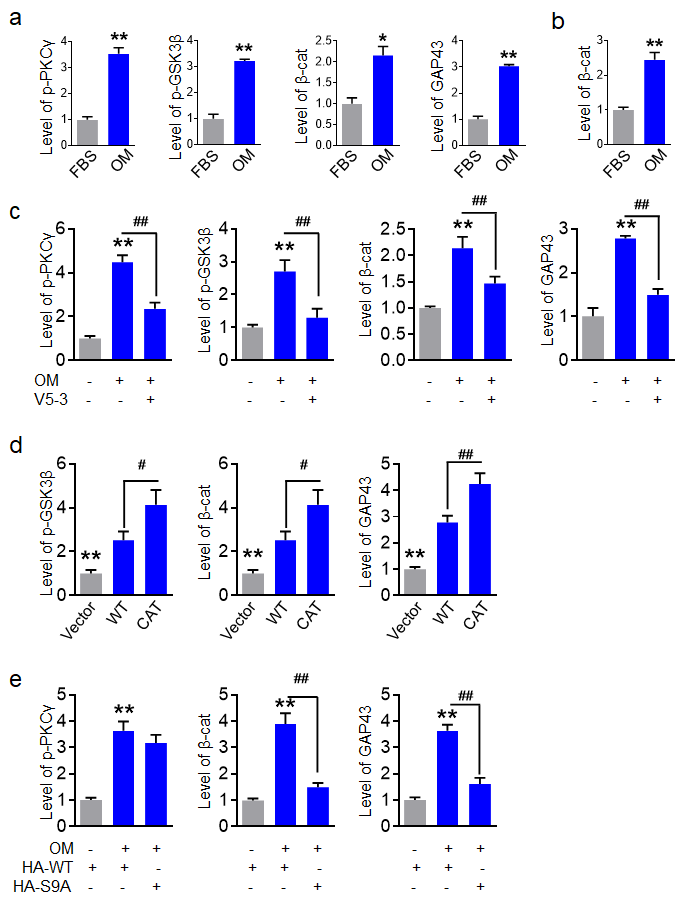
**

**Supplemental Figure S5. Quantification of indicated protein with Western blot in Fig. 5 a-c, e, f.**

Data are expressed as mean ± SD (n = 3 individual experiments in each group) and compared by Student’s *t-*test (a,b) or one-way ANOVA (c-e) (*p < 0.05, **p < 0.01, ^#^p < 0.05, ^##^p < 0.01 vs indicated group).


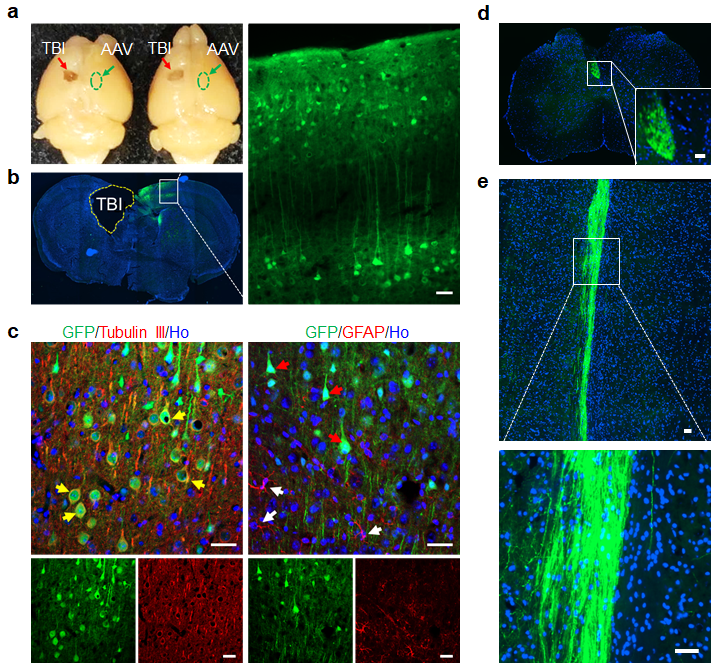


**Supplemental Figure S6**. **rAAV2/9-GFP mediated transduction specifically labels cortical neurons and CST.**

(a) Traumatic brain injury model. Red arrow showed a hole in left side of mouse brain 35 days post-TBI. Green circle represented the rAAV2/9-GFP injected area. (b) Brain sections were stained with Hoechst showed rAAV2/9-GFP was successfully infected in the mouse right cortex. The dotted line demarcates the injured area. Enlarged inset shows the efficiency of viral infection in the cortex (right panel). (c) rAAV2/9-GFP specifically infected cortical neurons. Tissue sections containing a GFP-positive area were stained with Tubulin III or GFAP. Left panel shows most GFP^+^ cells were Tubulin III positive (Yellow arrows indicate the representative neurons). Right panel shows that GFP^+^ cells were GFAP negative (Red arrows represent GFP^+^ cells and white arrows represent GFAP positive cells). (d) Cervical spinal cord section stained with Hoechst indicated rAAV2/9-GFP infected the left dorsal CST. (e) Horizontal section of cervical spinal cord showed the left CST was marked with GFP. Scale bars, 40 μm.

**
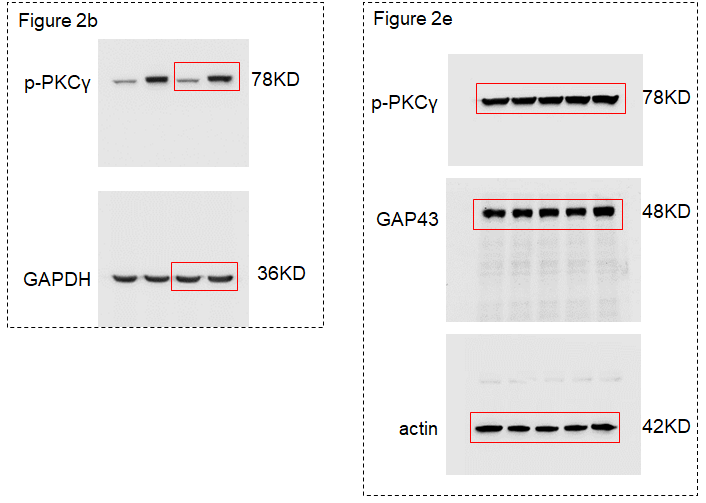
**

**
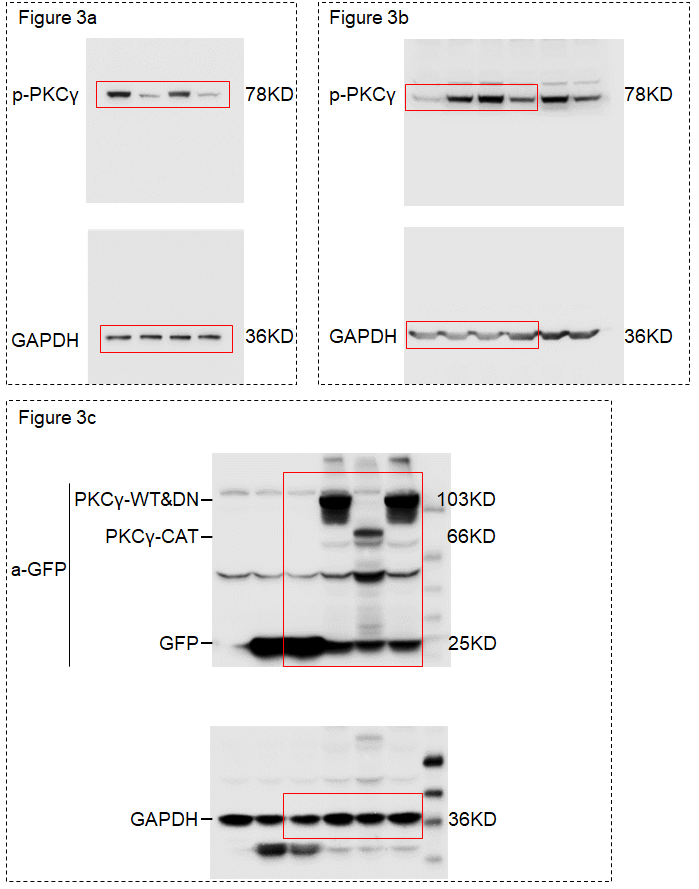
**

**
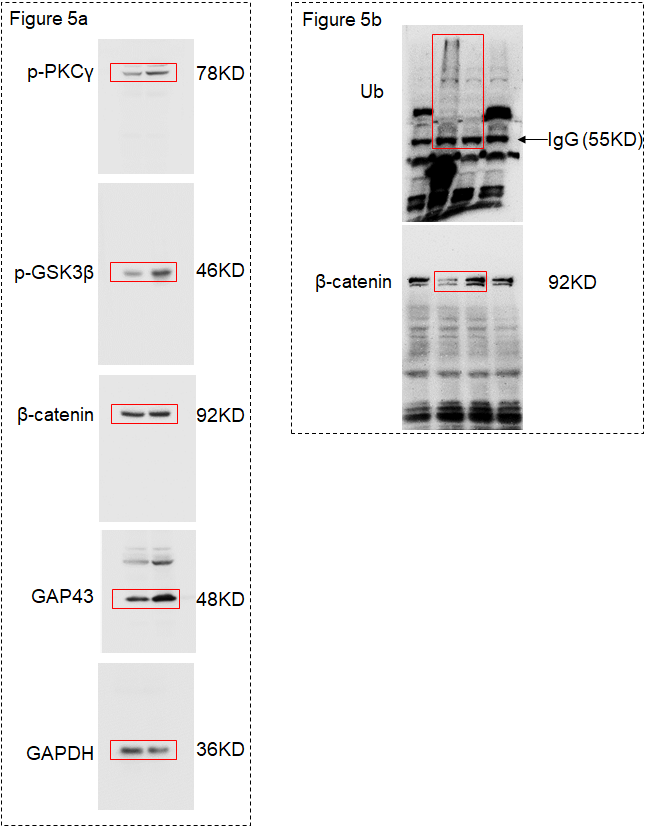
**

**
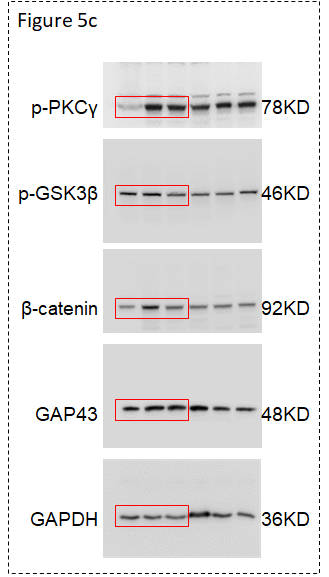
**

**
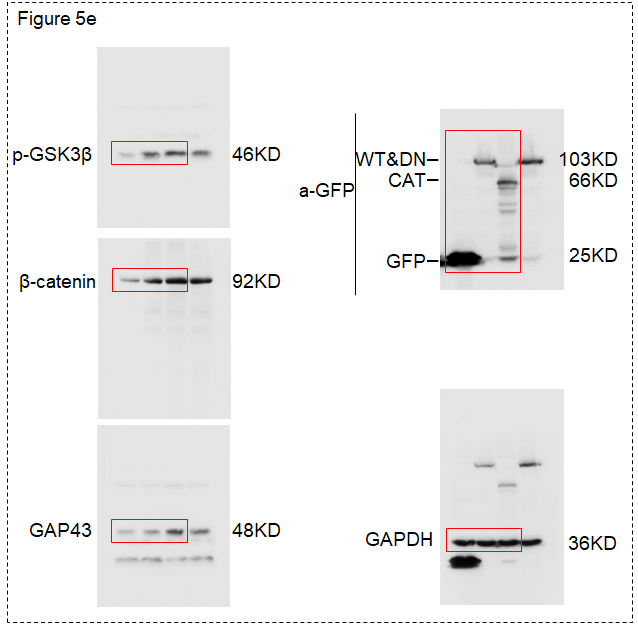
**

**
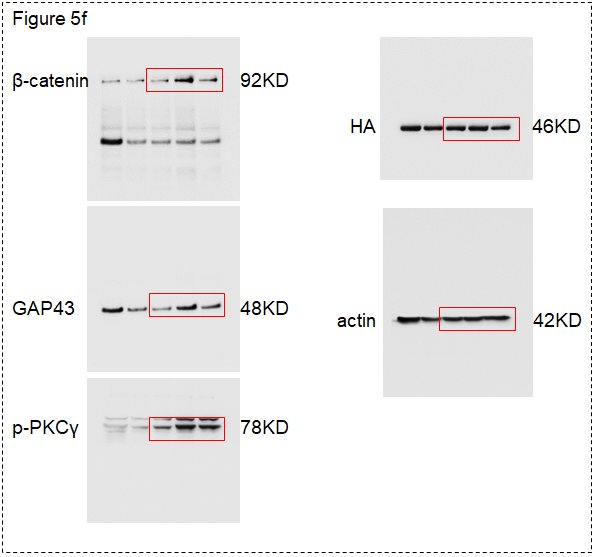
**

**
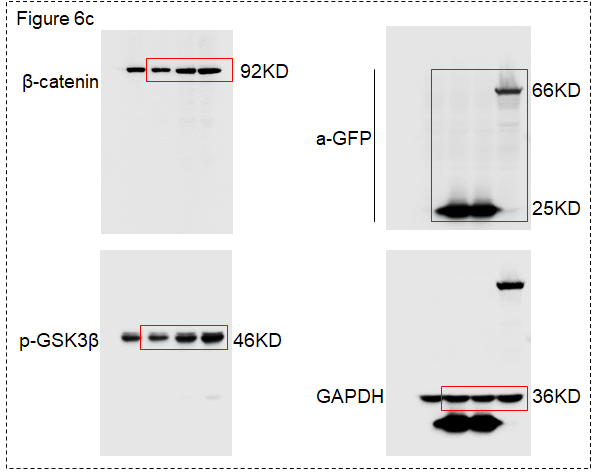
**

**
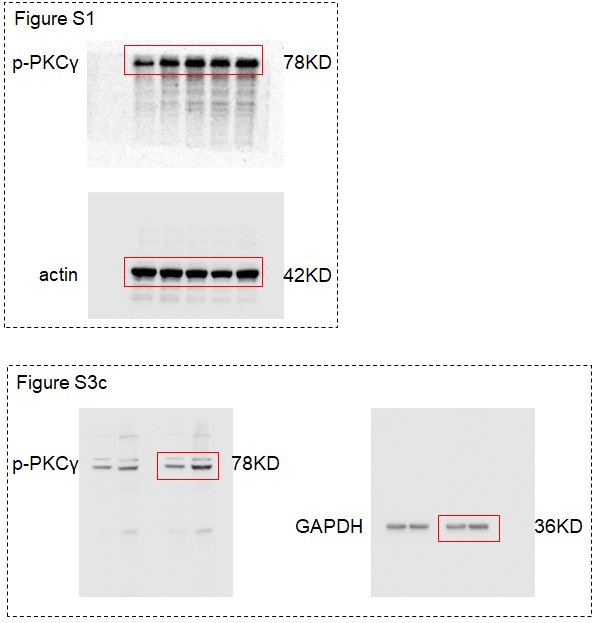
**

**Supplementary Table 1. List of used antibodies**

| **Antigen** | **Primary Antibody** | **Dilution** |
| --- | --- | --- |
| p-PKCγ | Abcam Cat# ab109539；  Rabbit polyclonal | 1:100 for IF;  1:1000 for WB |
| Tubulin III | R&D Systems Cat# MAB1195;  Mouse monoclonal | 1:400 for IF; |
| Tubulin III | Abcam Cat# ab18207;  Rabbit polyclonal | 1:200 for IF |
| MAP2 | Thermo Fisher Scientific Cat# MA5-12826; Mouse monoclonal | 1:100 for IF |
| GFAP | Millipore Cat# MAB360;  Mouse monoclonal | 1:200 for IF |
| Nestin | Abcam Cat# ab6142；  Mouse monoclonal | 1:100 for IF |
| SMI-31 | Millipore Cat# NE1022-100UL;  Mouse monoclonal | 1:1000 for IHC |
| MAG | Thermo Fisher Scientific Cat# 34-6200;  Rabbit polyclonal | 1:200 for IF |
| p-GSK3β(Ser9) | Cell Signaling Technology Cat# 9323;  Rabbit polyclonal | 1:1000 for WB |
| β-catenin | Abmart Cat# M24002;  Mouse monoclonal | 1:1000 for WB;  1:100 for IP |
| GAP43 | Abcam Cat# ab75810;  Rabbit polyclonal | 1:2000 for WB |
| Ub | Abcam Cat# ab7780;  Mouse monoclonal | 1:1000 for WB |
| GAPDH | Thermo Fisher Scientific Cat# MA5-15738-1MG; Mouse monoclonal | 1:5000 for WB |
| β-actin | Thermo Fisher Scientific Cat# MA5-15739-1MG; Mouse monoclonal | 1:2000 for WB |
| GFP-tag | Abcam Cat# ab1218;  Mouse monoclonal | 1:5000 for WB |
| HA-tag | Abmart Cat# M20003;  Mouse monoclonal | 1:5000 for WB  1:200 for IF |
